# Effects of Alpha-Lactalbumin or Whey Protein Isolate on Muscle Damage, Muscle Pain, and Mood States Following Prolonged Strenuous Endurance Exercise

**DOI:** 10.3389/fphys.2017.00754

**Published:** 2017-09-29

**Authors:** Lu Qin, Stephen H. S. Wong, Feng-Hua Sun, Yu Huang, Sinead Sheridan, Cindy H. P. Sit

**Affiliations:** ^1^Department of Sports Science and Physical Education, The Chinese University of Hong Kong, Hong Kong, Hong Kong; ^2^Department of Health and Physical Education, The Education University of Hong Kong, Tai Po, Hong Kong; ^3^School of Biomedical Sciences and Li Ka Shing Institute of Health Sciences, Institute of Vascular Medicine, The Chinese University of Hong Kong, Shatin, Hong Kong; ^4^Li Ka Shing Faculty of Medicine, School of Public Health, The University of Hong Kong, Pokfulam, Hong Kong

**Keywords:** nutrition, running, muscle recovery, stress, affect

## Abstract

This study compared the effect of alpha-lactalbumin and whey protein on muscle damage, muscle pain, and mood states during short term recovery following strenuous prolonged exercise. In a two-stage crossover counterbalanced design, 12 endurance male runners were recruited (age: 30.4 ± 2.8 year, height: 172.7 ± 5.6 cm, body mass: 66.7 ± 6.5 kg, VO_2max_: 58.0 ± 6.9 ml/kg^−^/min), ran for 90 min at 70% VO_2max_, and followed by a 4-h recovery. Two treatments (carbohydrate+alpha-lactalbumin, CA; carbohydrate+whey protein isolate, CW) were applied during the main trials. During the first 2-h of recovery, CHO was served at the rate of 0.66 g/kg/h and PRO at 0.34 g/kg/h every 30 min. Creatine kinase (CK), interleukin-6 (IL-6), salivary cortisol, rating of muscle pain, pressure pain threshold (PPT), and mood states were evaluated before (Pre-ex), immediately (Post-ex0), 2 h (Post-ex2h) and 4 h (Post-ex4h) after exercise. 24 h after exercise (Post-ex24h), CK and IL-6, muscle pain, and PPT were evaluated. Compared with Pre-ex, Post-ex24h CK was higher in both trials of CA (398.16 ± 41.37 vs. 184.77 ± 22.68 IU/L, *P* = 0.039) and CW (418.17 ± 67.86 vs. 202.41 ± 22.26 IU/L, *P* = 0.037). IL-6 was also higher than Pre-ex at Post-ex0 and Post-ex2h in trials of CA (Post-ex0 vs. Pre-ex0: 7.87 ± 0.74 vs. 1.69 ± 0.23, *P* < 0.01; Post-ex2h vs. Pre-ex0: 5.39 ± 0.88 vs. 1.69 ± 0.23, *P* = 0.02) and CW (Post-ex0 vs. Pre-ex0: 8.63 ± 1.06 vs. 1.59 ± 0.19, *P* < 0.01; Post-ex2h vs. Pre-ex0: 5.75 ± 1.33 vs. 1.59 ± 0.19, *P* < 0.01). No difference was found in CK and IL-6 between two trials at all time points (all *P* > 0.05). Compared with Pre-ex0, salivary cortisol was elevated at Post-ex0 in both trials (CA: 0.96 ± 0.13 vs. 0.41 ± 0.05 ng/ml, *P* < 0.01; CW: 1.15 ± 0.18 vs. 0.43 ± 0.06 ng/ml, *P* < 0.01) and was lower at Post-ex24h than Pre-ex in CA trial (0.17 ± 0.02 vs. 0.41 ± 0.05 ng/ml, *P* < 0.01). Compared with CW, PPT was higher at Post-2h in CA trial (31.55 ± 3.09 vs. 26.99 ± 2.32 N/cm^2^, *P* < 0.01). Compared with Post-ex0, feeling of fatigue was lower at Post-ex2h (*P* = 0.014) and Post-ex4h (*P* < 0.01) in CA, while it was lower at Post-ex4h (*P* = 0.038) in CW. Compared with CW, feeling of fatigue was marginally lower in the CA trial at Post-ex2h (*P* = 0.056). In conclusion, compared with the co-ingestion of CHO and whey PRO isolate, co-ingestion of CHO and alpha-lactalbumin reduced sensitivity to the muscle pain, attenuated feeling of fatigue and was more beneficial to reduce the feeling of fatigue and cortisol responses during 4-h recovery following 90-min running at 70% VO_2max_.

## Introduction

Prolonged strenuous exercise elicits a series of hemostatic alternations including metabolite accumulation (Tanaka et al., 1983), muscle glycogen depletion (Ivy et al., 2002), and muscle damage (Saunders et al., 2004). Those responses may result in muscle pain (Cheung et al., 2003; Dannecker and Koltyn, 2014). The perception of pain is transferred to the central nervous system evoking mood disturbance (Shacham et al., 1984). Both the above-mentioned disturbances in body homeostasis and mood states form stressors from perspectives of physiology and psychology and therefore induce stress in athletes (Goldstein and Kopin, 2007). In order to maintain hemostasis and facilitate the stress adaptation, the hypothalamus-pituitary-adrenal (HPA) axis is activated during exercise (Luger et al., 1987). This results in the increasing concentration of stress hormones, i.e., cortisol (Bachi et al., 2015). Inappropriate or inadequate recovery during the short term interval (2–8 h) may induce a series of negative impacts physiologically and psychologically including constant accumulation of cortisol (Meeusen et al., 2010) and a persistent negative impacts on mood states (e.g., increasing feeling of fatigue and decreasing feeling of vigor) (Lane and Wilson, 2011).

Carbohydrate (CHO) is one of the most utilized macronutrients in post-exercise short term recovery. The incorporation of protein (PRO) into post-exercise recovery nutrition, i.e., the co-ingestion of CHO and PRO, has been shown to further accelerate post-exercise muscle glycogen synthesis when compared with CHO alone served at a suboptimal rate (<1–1.2 g/kg/h) (van Loon et al., 2000; Ivy et al., 2002; Burke et al., 2011). The insulin response can be effectively stimulated when the PRO ingestion amount is 0.3–0.5 g/kg/h (Ivy et al., 2002; Berardi et al., 2006), therefore enhances the glycogen synthesis. In addition to muscle glycogen synthesis, the co-ingestion of protein, and CHO has been shown to increase the rate of muscle protein synthesis and improve whole body protein balance compared to the ingestion of CHO alone during recovery from endurance exercise (Howarth et al., 2009). In addition, the co-ingestion of CHO and PRO has also been reported to be more superior than the CHO alone in attenuating biomarkers for muscle damage (i.e., creatine kinase, CK) and muscle pain during post-exercise recovery of prolonged strenuous exercise (Saunders et al., 2004, 2009; Millard-Stafford et al., 2005; Hansen et al., 2015).

Whey PRO, the most commonly used dietary PRO in post-exercise recovery, has been shown to be superior to other dietary PROs (e.g., casein, soy) in enhancing muscle protein re-synthesis (Tang et al., 2009; Burd et al., 2012). Alpha-lactalbumin, a type of whey derived PRO which has gained considerable research attention in the last two decades, possesses a similar postprandial and digestive characteristic but has higher tryptophan than whey PRO. As tryptophan is the precursor to serotonin, this change suggested an increasing synthesis of serotonin in the central nervous system (CNS) (Markus et al., 2000, 2005). An enhancement of mood states with a lower feeling of depression, and a decreasing of cortisol has been reported after experimental stress following the ingestion of alpha-lactalbumin (Markus et al., 2000).

To date, few studies have reported the application of alpha-lactalbumin in exercise situation. In one of our previous studies (Qin et al., 2017), co-ingestion of alpha-lactabumin induced similar metabolic responses as whey PRO isolate in terms of blood glucose, free fatty acids, and insulin during 90 min running at 70% VO_2max_ in trained male runners. An enhancement of general core affect (pleasure-displeasure), which was associated with a decreased rating in muscle pain and plasma cortisol concentration, was also found following the ingestion of alpha-lactalbumin. However, little is still known about the effect of alpha-lactalbumin on muscle damage, muscle pain, and mood states during the short term recovery following strenuous prolonged endurance exercise. The purpose of this study was therefore to compare the effect of alpha-lactalbumin with whey PRO isolate on muscle damage, muscle pain, and mood states during short term recovery (4 h) following strenuous prolonged running. It was hypothesized that the ingestion of alpha-lactalbumin during short term recovery from prolonged strenuous exercise would be superior to whey PRO in attenuating muscle pain and enhancing mood states.

## Methods

### Ethics statement

All procedures used in this study followed the institutional guidelines in the Declaration of Helsinki and were approved by the Ethics Committee of the Chinese University of Hong Kong. The participants were informed of the procedures and the potential risks of the study and were free to ask questions during the briefing. They were then asked to provide written informed consent.

### Participants and experiment control

The inclusion criteria for participants were (1) young males (18–35 years); who (2) had participated in regular distance exercise training for >2 years; and (3) had completed at least one race longer than 10 km. The exclusion criteria were (1) had a current cardiovascular, respiratory, or endocrine system disorder, or a mental health disorder; (2) had an injury or surgery on the leg or back within the past 2 months; (3) had an allergy to any type of food or medication, especially whey protein, milk, or peanuts; (4) had taken antidepressant drugs in the previous 3 months; and (5) had a particular fear of seeing white coats, needles, syringes, or blood. Twelve healthy participants (age: 30.4 ± 2.8 year, height: 172.7 ± 5.6 cm, body mass: 66.7 ± 6.5 kg, VO_2max_: 58.0 ± 6.9 ml/kg/min; length of endurance exercise history: 8.5 ± 1.6 years) were eventually recruited. The recruited participants completed four separate tests (one VO_2max_ test, one familiarization test, and two main trials separated by a wash-out period of 7–21 days). Strenuous exercise, caffeine, alcohol, and nicotine were avoided for 48 h before each experimental day. In order to ensure an overnight fasting state for 10–12 h, participants refrained from any food and liquid ingestion except water after 8:00 p.m. before the main trial days. During the washout period between the main trials, information about the participants' dietary content, physical activity, and life events (disease or injury, examination dates for students, stressful work, and life events) was obtained.

### Experiment protocol

#### Main trials and drinking protocol

For each main trial, participants reported to the same experimental site between 07:00 and 07:30 a.m. A sip of water was provided to rinse the mouth for the preparation of saliva collection. Approximately 30–45 min was allowed for changing into sports gear, emptying the bladder, and colon, and then sitting quietly for resting. Participants then conducted the exercise by running on the treadmill for 90 min at a constant speed equivalent to 70% of their individual VO_2max_. Pure water was served at 5 ml/kg immediately before exercise and at 2.5 ml/kg at 30, 60 min during exercise. After the exercise, a short shower was allowed. Participants came back to the lab afterward for recovery. During the 4-h recovery, two prescribed drinks were arranged in a double-blinded counterbalanced order. Two treatments (CHO+alpha-lactalbumin, CA; CHO+Whey Protein Isolated, CW) were applied. Whey protein isolate (WPI) and alpha-lactalbumin (containing <1.0% sunflower lecithin to aid dispersibility) were purchased from the same manufacturer (Davisco®, Le Sueur, MN, USA). We did not involve the CHO or placebo control due to the efficacy of whey PRO during the short term recovery had already been evidenced in previous studies, and also to make it comparable between those two treatments in the peripheral metabolic status as well as the amino acids availability to the peripheral muscle. Protein powder was dissolved and served along with a 6.6% CHO solution (w/v%, provided by Porcari®, Otsuka Pharmaceutical Co., Ltd., Japan) and the amount of fluid intake was determined as 10 ml/kg. During the first 2 h of the recovery, CHO was served at the rate of 0.66 g/kg/h while PRO at 0.34 g/kg/h. The total amount of fluid and PRO powder were divided into six equal portions and served at every 30 min. Body weight measurement (MC-780U, TANITA®, USA), capillary blood and saliva sampling, scales evaluation (abdominal discomfort, thirst, muscle pain, perceived exertion, and mood states) and pressure pain threshold (PPT) assessment were conducted pre- (Pre-ex) and post-exercise (Post-ex0), at every 2 h during the recovery (Post-ex2h and Post-ex4h). In the next morning of the experimental day (Post-ex24h), participants were asked to visit the lab for a single set of capillary blood sampling, scale evaluation for muscle pain and PPT assessment. In order to control the macronutrients intake before the next morning's visit, standardized meals (Energy content: 17.97 ± 0.45 kcal/kg; 57% CHO, 15% PRO, 28% FAT) were provided during the rest of the main trial day. No other food and energy-contained liquid drinks were allowed.

#### Blood measurements

After facilitating blood flow to sampling site by submerging their hands in warm water, the capillary blood sample was obtained from the participants' fingertips. For each sampling time, 1 ml of capillary blood sample was withdrawn with two 600 μl multivettes (Multivettes® 600K3E, Sarstedt AG & Co., Germany). Blood samples were centrifuged (Primo™ R, Biofuge®, Heraeus Ltd., Hanrau, Germany) for 10 min at 3500 rpm at 4°. K^+^-EDTA anti-clotting plasma were collected in Eppendorf tubes and stored at −80° in an ultra-low temperature freezer (Model MDFU52V, Sanyo™, Sanyo Electric Co., Ltd., Japan) before analysis. Blood glucose (BG) was measured using YSI 1500 glucose analyzer (Yellow Springs Instruments, Yellow Springs, OH). Lactic acid (LA) was measured using an YSI 1500 lactate analyzer (Yellow Springs Instruments, Yellow Springs, OH). CK and hemoglobin was evaluated with a reflectance photometer (Reflontron® Plus system, Roche Diagnostics Ltd., Switzerland). Plasma interleukin-6 (IL-6) was evaluated by enzyme linked immunosorbent assay (ELISA) kit (Quantikine® HS, R&D Systems Ltd., UK).

#### Salivary cortisol

A piece of Salivabio oral swab (SOS) (Salmeterics®, USA) was positioned under the front of the tongue to collect saliva. The SOS was then removed from the oral capacity and inserted into the barrel of a 5 ml syringe. Approximately 0.5 ml of saliva sample was squeezed into an Eppendorf tube and stored at −80° in an ultra-low temperature freezer (Model MDFU52V, Sanyo™, Sanyo Electric Co., Ltd. Japan) for further determination of cortisol concentration. The salivary cortisol concentration was determined using ELISA kit (Salmeterics®, USA).

#### Muscle pain, abdominal discomfort, thirst, and pressure pain threshold

Muscle pain, abdominal discomfort, thirst, and perceived exertion were all measured by rating scales that have been previously applied in the same experiment site (Li et al., 2015; Qin et al., 2017). Muscle pain was evaluated using an 11-point pain intensity scale ranging from 0 (no pain at all) to 10 (extremely intense pain, almost unbearable). Abdominal discomfort (AD) was evaluated based on the discomfort sensation of gastrointestinal system, i.e., stomachache, with an 11-point abdominal discomfort scale ranging from 0 (completely comfortable) to 10 (unbearable pain). Thirst was evaluated with the rating scale ranging from 0 (not thirsty) to 10 (very, very thirsty). PPT suggested the pain sensitivity to the peripheral stimulus and was evaluated with the algometer (FORCE ONE™- MODEL FDIX, Wagner instrument, USA). During the evaluation, participants placed both of their legs in a supine position without any muscle contraction or movement. A round metal probe with the diameter of 1 cm was equipped on one end of the algometer. When assessing the PPT, the probe was placed on the middle part of the rectus femoris and the force gradually increased by 3 N/cm^2^ per second. Each participant indicated information by saying “stop” or “okay” to the experimenter when the feeling of pressure turned to slightly weak pain. The force value was recorded afterward. Assessment for each leg was repeated for three times and the PPT for the particular leg was determined as the average value of the two closest numbers. The average number for the PPT of the two legs was considered as the PPT of the leg muscle for this assessment (Astokorki and Mauger, 2017). The middle part of the rectus femoris was identified as the middle point of the line linked between the anterior superior iliac spine and the patella. The middle part was marked with a water-resistant marker pen to ensure the identical part for each evaluation.

#### Chinese version of brunel mood scale (BRUMS-C)

The Chinese version of Brunel Mood Scale (BRUMS-C) was applied to determine the mood states in terms of specific dimensions. BRUMS-C has been validated in a previous study (Zhang et al., 2014). Each item was rated in response to a 5-point scale that ranges from 0 “not at all” to 4 “extremely,” with four items in dimension of Anger, four in Confusion, four in Fatigue, three in Tension, and four in Vigor. The raw score of the scale for each dimension was calculated and total mood disturbance (TMD) was determined by the sum score of negative dimension (anger, confusion, tension, depression, fatigue) substrate by the score of positive dimension (vigor).

### Statistical analyses

All continuous data were analyzed with IBM SPSS 20.0 and are presented as Mean ± Standard Error of the Mean (SEM). Cross-over data for repeated measures was analyzed using two-way repeated measures ANOVA. Treatment (CA, CW) and time (Pre-ex, Post-ex0, 2h, 4h, and 24h) were involved as within-subject factors, while trial order (the order sequence that the subject received treatments) as between-subject factor. Simple effect analysis with Bonferroni *post-hoc* was applied to determine the difference among treatment or time. Bivariate Pearson correlation was applied to examine the relationship between key physiological and scale measurements. The correlation strength was determined with the absolute value of Pearson's *r* (very weak: *r* = 0–0.19; weak: *r* = 0.20–0.39; moderate: *r* = 0.41–0.59; strong: *r* = 0.60–0.79; very strong: *r* = 0.80–1.00) (Evan, 1995). The significance level was determined as α = 0.05.

## Results

### Creatine kinase (CK) and interleukin-6 (IL-6)

**Figure 1:**
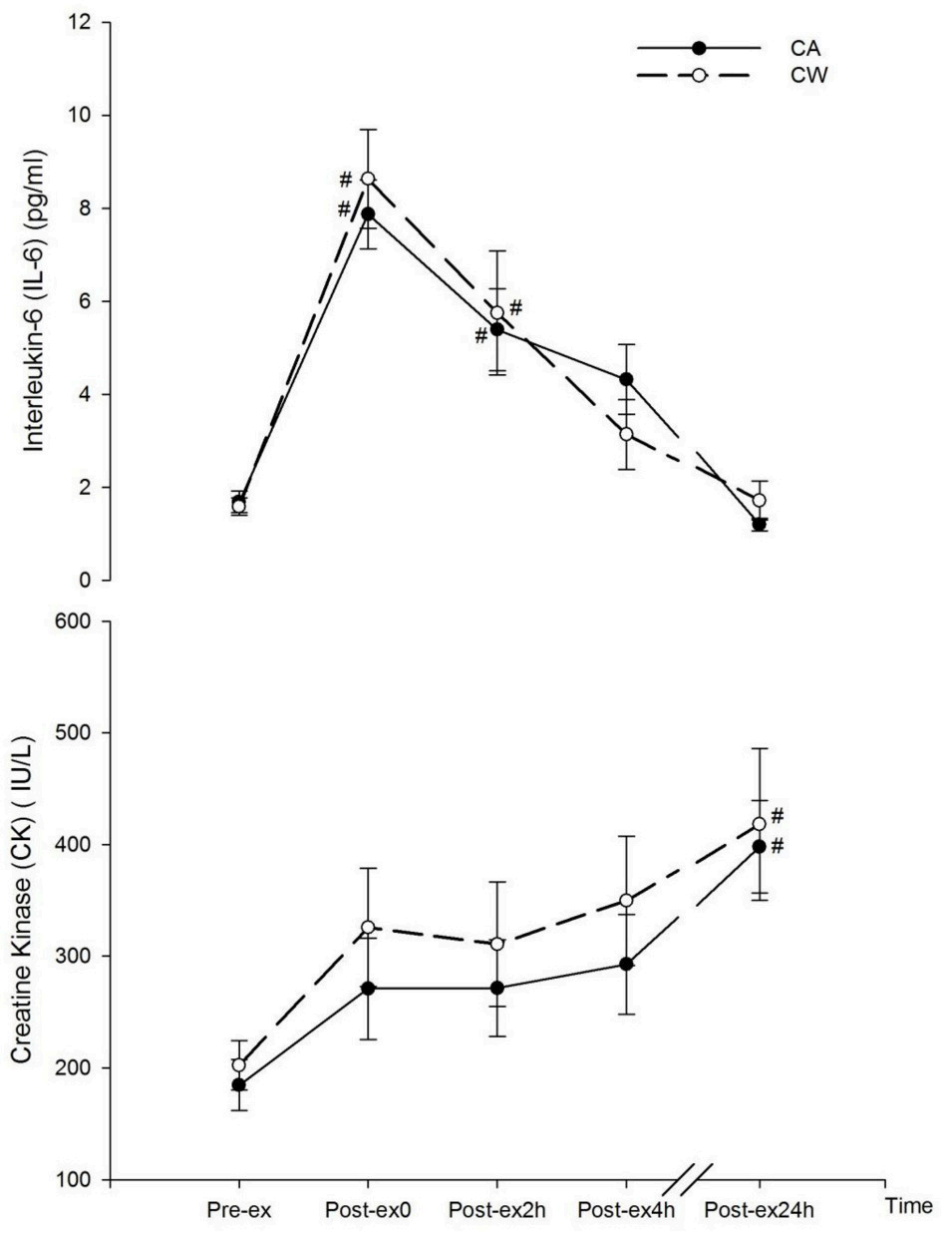
Changes in CK and IL-6 following main trials (^#^vs. Pre-ex0: *P* < 0.01). CA, CHO+Alpha-lactalbumin; CW, CHO+Whey PRO.

Compared with Pre-ex0, CK increased at Post-ex24h in CA (398.16 ± 41.37 vs. 184.77 ± 22.68 IU/L, *P* = 0.039) and CW (418.17 ± 67.86 vs. 202.41 ± 22.26 IU/L, *P* = 0.037). IL-6 was also higher than Pre-ex at Post-ex0 and Post-ex2h in CA (Post-ex0 and Post-ex2h vs. Pre-ex0: 7.87 ± 0.74 and 5.39 ± 0.88 vs. 1.69 ± 0.23, *P* < 0.05) and CW (Post-ex0 and Post-ex2h vs. Pre-ex0: 8.63 ± 1.06 and 5.75 ± 1.33 vs. 1.59 ± 0.19, *P* < 0.01) (Figure 1).

### Blood glucose (BG) and lactic acid (LA)

**Figure 2:**
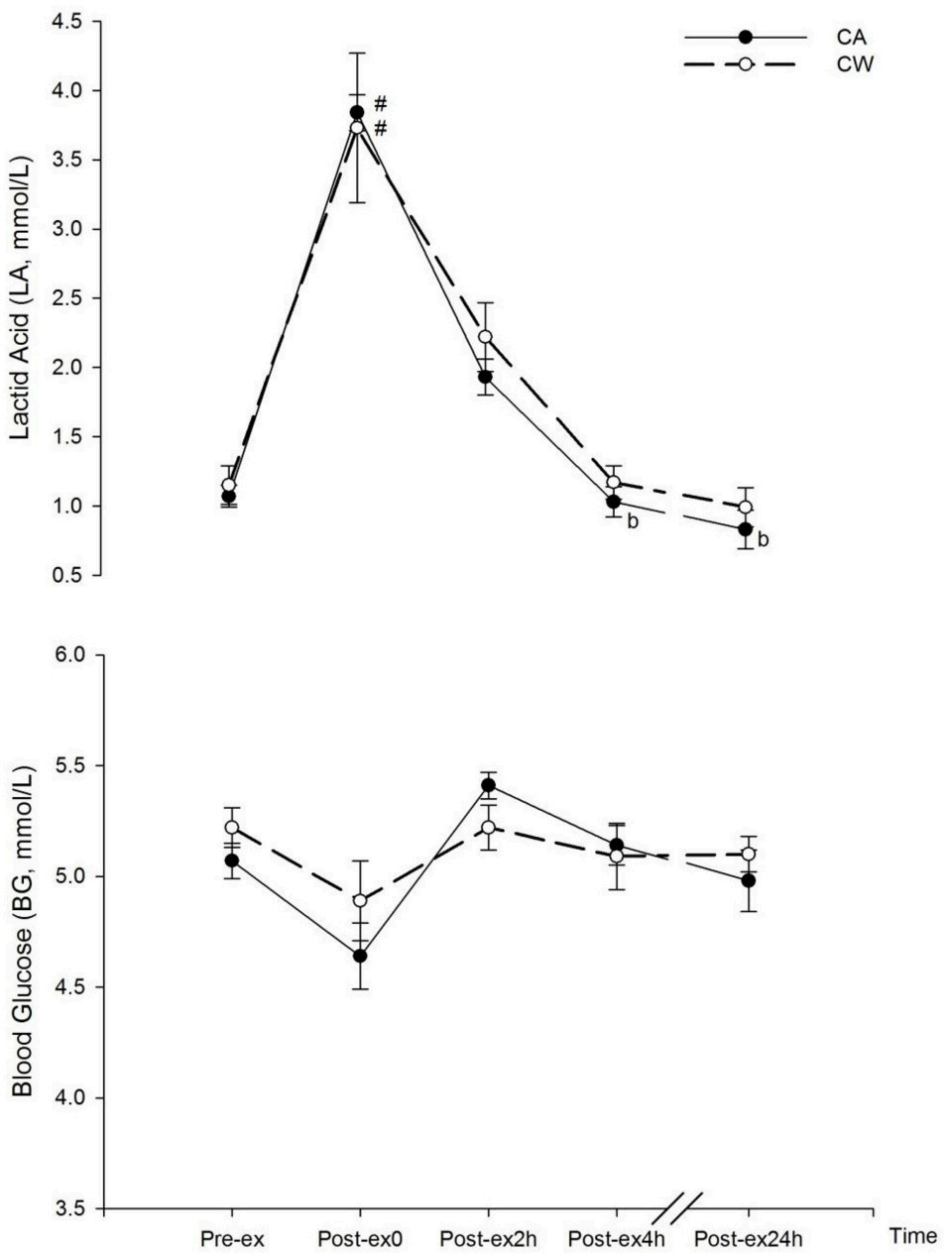
Changes in BG and LA following main trials (^#^vs. Pre-ex0: *P* < 0.05; ^*b*^vs. Post-ex0: *P* < 0.05). CA, CHO+Alpha-lactalbumin; CW, CHO+Whey PRO.

Compared with the baseline (Pre-ex), LA marginally increased after exercise (Post-ex0) in CA (3.84 ± 0.48 vs. 1.07 ± 0.08 mmol/l, *P* = 0.053) and CW (3.73 ± 0.54 vs. 1.15 ± 0.14 mmol/l, *P* = 0.056). Compared with Pre-ex, LA decreased at Post-ex4h following the ingestion of CA (1.03 ± 0.11 vs. 3.84 ± 0.48 mmol/l, *P* = 0.039)(Figure 2).

### Body weight (BW) and hemoglobin (Hb)

Compared with Pre-ex, BW decreased at Post-ex0 in CA (*P* < 0.01) and CW (*P* < 0.01). Compared with Pre-ex, Hb concentration was higher in CA (*P* < 0.01) and CW (*P* < 0.01) (Table 1).

**Table 1:** Change of body weight and hemoglobin following main trials.

	**Pre-ex**	**Post-ex0**	**Post-ex2h**	**Post-ex4h**
**BW (kg)**				
CA	67.73 ± 1.84	66.43 ± 1.80^#^	67.49 ± 1.81	67.62 ± 1.82
CW	67.29 ± 1.82	65.92 ± 1.85^#^	67.00 ± 1.83	67.23 ± 1.83
**Hb (g/L)**				
CA	13.93 ± 0.42	14.60 ± 0.35^#^	14.19 ± 0.27	14.12 ± 0.38
CW	14.25 ± 0.39	14.76 ± 0.38^#^	14.26 ± 0.41	14.05 ± 0.44

*CA, CHO+Alpha-lactalbumin; CW, CHO+Whey PRO; BW, Body weight; Hb, hemoglobin*.

# *vs. Pre-ex0, P < 0.01*.

### Muscle pain, abdominal discomfort (AD), thirst, and pressure pain threshold (PPT)

Compared with Pre-ex0, subjective rating of muscle pain, AD, and thirst increased at Post-ex0 (all *P* < 0.01) (Table 2). Compared with CW, PPT was higher at Post-2h in CA trial (31.55 ± 3.09 vs. 26.99 ± 2.32 N/cm^2^, *P* < 0.01) (Figure 3).

**Table 2:** Muscle pain, abdominal discomfort, and thirst following treatments (M ± SE).

	**Pre-ex**	**Post-ex0**	**Post-ex2h**	**Post-ex4h**	**Post-ex24h**
**PAIN**					
CA	0.45 ± 0.22	4.79 ± 0.72^#^	1.33 ± 0.39	1.25 ± 0.34	1.45 ± 0.60
CW	0.45 ± 0.17	4.58 ± 0.68^#^	1.50 ± 0.35	1.16 ± 0.34	1.25 ± 0.38
**AD**					
CA	0.75 ± 0.13	4.91 ± 0.63^#^	2.16 ± 0.47	1.58 ± 0.48	–
CW	0.83 ± 0.21	5.25 ± 0.67^#^	2.33 ± 0.60	2.16 ± 0.70	–
**THIRST**					
CA	0.91 ± 0.23	5.41 ± 0.57^#^	1.25 ± 0.44	1.16 ± 0.32	–
CW	1.25 ± 0.37	5.33 ± 0.62^#^	1.66 ± 0.54	1.08 ± 0.43	–

*CA, CHO+Alpha-lactalbumin; CW, CHO+Whey PRO; Pain, Rating of muscle pain; AD, Rating of abdominal discomfort; Thirst, Rating of thirst*.

# *vs. Pre-ex, P < 0.01*.

**Figure 3:**
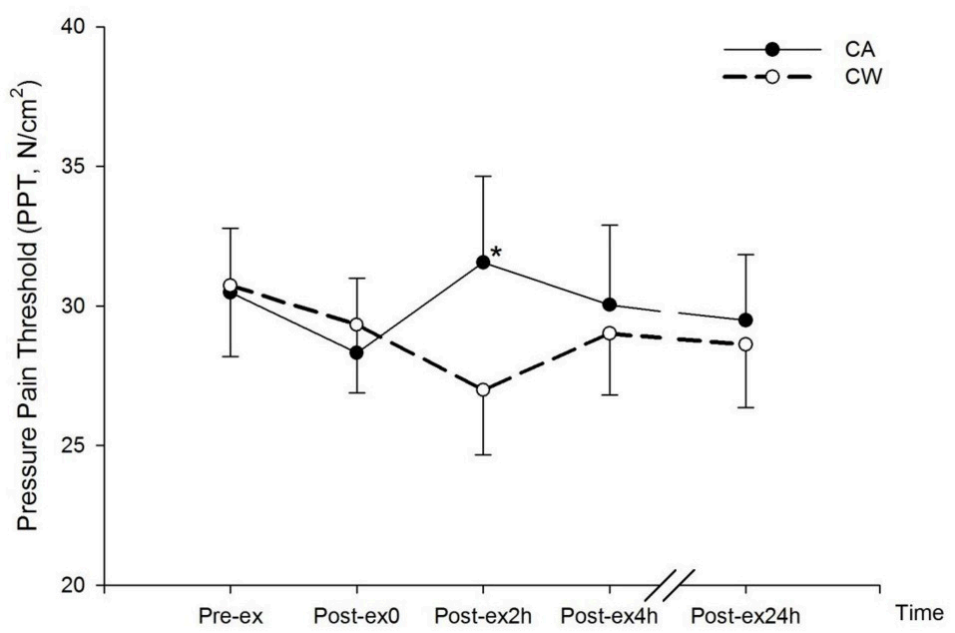
Changes in Pressure Pain Threshold (PPT) following main trials. (^*^vs. CW, *P* < 0.01; CA, CHO+Alpha-lactalbumin; CW, CHO+Whey PRO).

### Mood states and salivary cortisol

**Figure 4:**
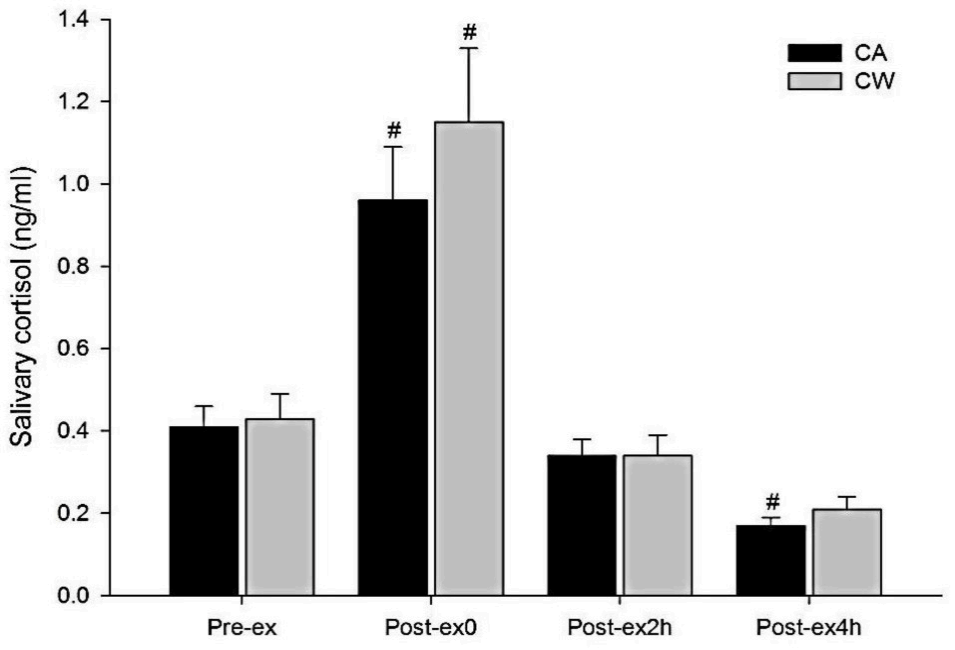
Changes in salivary cortisol following main trials (^#^vs. Pre-ex: *P* < 0.01, CA, CHO+Alpha-lactalbumin; CW, CHO+Whey PRO).

Compared with Pre-ex0, feelings of fatigue and TMD were higher at Post-ex0 in CA and CW (all *P* < 0.01). Compared with Post-ex0, feeling of fatigue was lower at Post-ex2h (*P* = 0.014) and Post-ex4h (*P* < 0.01) in CA, while it was lower at Post-ex4h (*P* = 0.038) in CW. Compared with CW, feeling of fatigue was marginally lower in the CA at Post-ex2h (*P* = 0.056) (Table 3). Compared with Pre-ex, the salivary cortisol was higher at Post-ex0 in both trials of CA (0.96 ± 0.13 vs. 0.41 ± 0.05 ng/ml, *P* < 0.01) and CW (1.15 ± 0.18 vs. 0.43 ± 0.06 ng/ml, *P* < 0.01). At Post-ex4h, the salivary cortisol was lower than Pre-ex in CA trial (0.17 ± 0.02 vs. 0.41 ± 0.05 ng/ml, *P* < 0.01) (Figure 4).

**Table 3:** Change of mood states in both trials (M ± SE).

	**Pre-ex**	**Post-ex0**	**Post-ex2h**	**Post-ex4h**
**ANGER**				
CA	1.16 ± 0.74	2.17 ± 1.09	0.41 ± 0.34	0.41 ± 0.26
CW	0.00 ± 0.00	1.66 ± 0.72	0.25 ± 0.25	0.33 ± 0.19
**CONFUSION**				
CA	1.75 ± 0.51	3.42 ± 1.21	1.33 ± 0.52	1.08 ± 0.39
CW	1.67 ± 0.54	4.33 ± 1.35	0.83 ± 0.29	0.67 ± 0.28
**DEPRESSION**				
CA	1.50 ± 0.82	2.58 ± 1.13	0.67 ± 0.39	0.75 ± 0.41
CW	0.25 ± 0.13	2.50 ± 0.96	0.50 ± 0.42	0.50 ± 0.28
**FATIGUE**				
CA	3.83 ± 0.61	7.17 ± 1.07^##^	4.75 ± 0.95^a^^Δ^	3.16 ± 0.78^aa^
CW	3.33 ± 0.48	8.00 ± 1.61^##^	5.25 ± 1.10	4.41 ± 0.98^a^
**TENSION**				
CA	2.00 ± 0.71	2.50 ± 1.20	0.75 ± 0.46	0.33 ± 0.25
CW	1.00 ± 0.43	2.58 ± 1.02	0.33 ± 0.18	0.08 ± 0.08
**VIGOR**				
CA	4.08 ± 0.82	3.33 ± 0.67	3.75 ± 0.92	4.08 ± 0.47
CW	3.75 ± 0.68	3.58 ± 0.37	3.58 ± 0.28	3.16 ± 0.47
**TMD**				
CA	6.17 ± 3.11	14.50 ± 5.52^##^	4.16 ± 2.62	1.67 ± 2.08
CW	5.50 ± 1.23	15.40 ± 5.04^##^	3.58 ± 1.52	2.83 ± 1.34

CA, CHO+Alpha-lactalbumin; CW, CHO+Whey PRO; TMD, total mood disturbance;

## vs. Pre-ex, P < 0.01;

Δ vs. CW, P = 0.056;

a vs. Post-ex0, P < 0.05;

aa *vs. Post-ex0, P < 0.01*.

### Correlation analysis

Correlation analysis was conducted among the measurements of muscle damage (CK, IL-6), muscle pain and dimensions of mood states from Pre-ex to Post-ex4h (Table 4). Weak negative correlations were found between CK and feelings of depression (*r* = −0.227, *P* < 0.05) and tension (*r* = −0.228, *P* < 0.05). A moderate positive correlation was found between IL-6 and muscle pain (*r* = 0.452, *P* < 0.01), feeling of fatigue (*r* = 0.298, *P* < 0.01), and TMD (*r* = 0.470, *P* < 0.01). Salivary cortisol concentration was moderately and positively correlated with muscle pain (*r* = 0.574, *P* < 0.01), all negative mood dimensions of anger (*r* = 0.369, *P* < 0.01), confusion (*r* = 0.473, *P* < 0.01), depression (*r* = 0.414, *P* < 0.01), fatigue (*r* = 0.390, *P* < 0.01), tension (*r* = 0.434, *P* < 0.01), and TMD (*r* = 0.470, *P* < 0.01). Strong and positive correlation was found between muscle pain and feeling of fatigue (*r* = 0.708, *P* < 0.01), confusion (*r* = 0.669, *P* < 0.01), and TMD (*r* = 0.709, *P* < 0.01). A moderate positive correlation with feelings of depression (*r* = 0.539, *P* < 0.01) and tension (*r* = 0.434, *P* < 0.01), while muscle pain was moderately and negatively correlated with PPT (*r* = 0.371, *P* < 0.01). PPT was moderately and negatively correlated with mood dimensions of anger (*r* = −0.342, *P* < 0.01), confusion (*r* = −0.445, *P* < 0.01), depression (*r* = −0.325, *P* < 0.01), fatigue (*r* = −0.462, *P* < 0.01), and TMD (*r* = −0.431, *P* < 0.01).

**Table 4:** Correlation among measurements of muscle damage, muscle pain, and mood states.

	**CK**	**IL6**	**Cortisol**	**Pain**	**PPT**	**Anger**	**Confusion**	**Depression**	**Fatigue**	**Tension**	**Vigor**	**TMD**
CK	–											
IL6	0.248^*^	–										
Cortisol	−0.090	0.391^**^	–									
Pain	−0.053	0.452^**^	0.574^**^	–								
PPT	−0.050	−0.012	−0.110	−0.371^**^	–							
Anger	−0.170	0.188	0.369^**^	0.526^**^	−0.342^**^	–						
Confusion	−0.192	0.161	0.473^**^	0.669^**^	−0.445^**^	0.702^**^	–					
Depression	−0.227^*^	0.140	0.414^**^	0.539^**^	−0.325^**^	0.903^**^	0.756^**^	–				
Fatigue	0.078	0.298^**^	0.390^**^	0.708^**^	−0.462^**^	0.462^**^	0.699^**^	0.503^**^	–			
Tension	−0.228^*^	0.124	0.434^**^	0.539^**^	−0.312^**^	0.823^**^	0.863^**^	0.860^**^	0.525^**^	–		
Vigor	0.153	0.032	−0.020	−0.137	−0.023	−0.033	−0.050	−0.084	−0.185	0.036	–	
TMD	−0.168	0.210^*^	0.470^**^	0.709^**^	−0.431^**^	0.831^**^	0.906^**^	0.876^**^	0.795^**^	0.874^**^	−0.262^**^	–

*CK, Creatine kinase; IL-6, Interleukin-6; PPT, Pressure pain threshold; TMD, Total mood disturbance*.

* P < 0.05;

** *P < 0.01*.

## Discussion

The main findings of the present study included: (1) In both trials of CA and CW, concentration of CK, IL-6, and cortisol increased following strenuous prolonged treadmill running at 70% VO _2max_. These responses were associated with increased muscle pain, ratings of negative mood dimension, and TMD; (2) Compared with CW, PPT was higher in CA trial at Post-ex2h; (3) Compared with CW, CA trial was more beneficial for reducing the feeling of fatigue during the 4-h's short term recovery; (4) In CA trial, cortisol concentration reduced at Post-ex4h.

CK is considered as one of the gold standard biomarkers to evaluate the muscle damage. It appears in the blood stream following the occurrence of muscle damage, and gradually increases to the peak level during the following 24–48 h (Saunders et al., 2004). In the present study, measurement of CK was used to evaluate the combined effect of both exercise-induced and the post exercise ingestion of the two nutritional treatments on muscle damage. In addition, muscle damage induces a series of inflammatory responses. Immunological cytokines such as IL-6 are rapidly relieved from the muscle tissue (Ostrowski et al., 1998). Although not necessarily associated with the damage in muscle, IL-6 could also be relieved from the contracting muscle and was found to be correlated with the exercise intensity (Ostrowski et al., 2000). Given the consideration that CK would not increase rapidly following acute exercise bout and the change of CK concentration could be the combined effect of exercise, nutrition supplementation, and the physical activity during the interval in the following 24 h, IL-6 was used to partly serve as one of the biomarkers for acute muscle damage following strenuous prolonged exercise and to determine whether the exercise intensity and muscle contraction was comparable between two trials (Ostrowski et al., 1998; Bernecker et al., 2013). In the present study, participants conducted two bouts of strenuous prolonged treadmill running at 70%VO_2max_ in two separate main trials. Immediately following the exercise (Post-ex0), there was no difference in IL-6 concentration between both trials, suggesting a similar acute muscle damage response which made it comparable to evaluate the effect of CA and CW ingestion on muscle damage following exercise (Figure 1). Consistent with previous studies (Ostrowski et al., 1998; Nieman et al., 2005), concentration of IL-6 increased in Post-ex0 and gradually decreased during the recovery (Figure 1), and the IL-6 was moderately associated with muscle pain (Table 4). This indicated muscle pain may occur partly due to the acute onset of exercise induced muscle damage. In association with the increasing muscle pain, mood disturbance occurred at the end of exercise (Post-ex0). Cortisol, which is associated with negative affect, increased following exercise as a result (Figure 4).

The beneficial effect of CHO and PRO supplementation in post-exercise recovery was partly facilitated by the stimulation of insulin secretion and therefore assists in the glycogen repletion as well as protein resynthesis (Millard-Stafford et al., 2005; Berardi et al., 2006). In addition, the timing of ingestion is also essential when evaluating the role of CHO and PRO supplementation in optimal recovery. Compared with ingesting CHO 2 h after exercise, the rate of glycogen re-synthesis was reported to be ~50% higher (7.7 vs. 4.1 μmol/g/h) when the CHO was ingested immediately after exercise (Ivy et al., 1988). A positive net protein balance (+60.5 μg/min/100 ml) was also reported when PRO ingestion immediate after exercise, while it was negative (−23.8 μg/min/100 ml) when PRO was ingested 3 h later (Levenhagen et al., 2001). In the present study, prescribed treatments (CA and CW) were served during the first 2 h of recovery. The amount of CHO and PRO were identical, whereas the types of PROs were different in these two treatments. Similar blood glucose responses (Figure 3) suggested similar CHO availability in both trials. Moreover, alpha-lactalbumin and whey PRO share the similar characteristics regarding to insulin responses (Mortensen et al., 2012) and large neutral amino acids (LNAA) profiles that are mainly untaken by the muscle fiber for protein resynthesize (Tipton et al., 2007). There was no difference between CA and CW trials in terms of both CK and IL-6 at Post-ex2h, Post-ex4h, and Post-ex24h (Figure 1). This suggested similar effect on muscle damage following the ingestion of CA and CW.

Muscle pain was associated with negative mood and TMD (Table 4). Before nutrition supplementation, muscle pain increased similarly between the two bouts of exercise in trials of CA and CW (Table 2). In a previous study (Qin et al., 2017), muscle pain was attenuated when CA was ingestion during exercise. However, muscle pain was not different between the two trials during the recovery following the ingestion of CA and CW. During short term recovery from exercise, participants sat rest with the supine position and result no external stimulus as during exercise. Rating of muscle pain was not necessarily increased. PPT assessment was therefore involved in order to achieve the external mechanical stimulation of muscle pain. At Post-ex2h, PPT was higher in CA trial than in CW trial (Figure 3). This indicated a lower pain sensitivity following the ingestion of CA than CW. Compared with whey PRO, alpha-lactalbumin possesses a higher amount of tryptophan. The total tryptophan to LNAA in plasma increased by 48–130% following the ingestion of ~20 grams of alpha-lactalbumin in participants in a non-fasting state (Markus et al., 2000, 2005). Although it was not directly evaluated, there should be a greater increase in total tryptophan following (45.61 ± 0.45) g alpha-lactalbumin ingestion in the present study under a fasting state. As serotonin synthesis in the hypothalamus is positively associated with total tryptophan concentration (Bloxam et al., 1980), it is reasonable to speculate that the serotonin availability in the CNS, including hypothalamus, increased following the ingestion of CA. As the hypothalamus is a crucial part for pain perception (Craig, 2002), increased serotonin availability in this area reduces the transmission of pain (Kupers et al., 2011). Pain sensitivity in the present study was therefore reduced following the ingestion of CA. However, as the tryptophan strictly relies on out resourced amino acid supplementation, serotonin availability was therefore gradually decreased after 2 h' supplementation. PPT in Post-ex4h and Post-ex24 was similar in both trials (Figure 3). This result suggests that a longer effect for pain attenuation can be achieved by extending the ingestion period.

A previous study (Moyen et al., 2015) showed that hydration status was positively associated with feeling of fatigue and negatively associated with feeling of vigor. Body weight and the concentration of hemoglobin were therefore evaluated as control variables to determine whether there was a difference in fluid loss and plasma volume between both trials. No treatment effects were found for these two variables (Table 1). This made it comparable when determining the difference between mood states in each of the two trials. Consistent with previous studies (Lane et al., 2005; Bachi et al., 2015), the feeling of fatigue increased following the exercise bout in the present study. Compared with immediately after the exercise (Post-ex0), feeling of fatigue was lower at 2 h post exercise (Post-ex2h) and 4 h post exercise (Post-ex4h) in CA, while it was lower at 4 h post exercise (Post-ex4h) in CW. In addition, feeling of fatigue in 2 h post exercise (Post-ex2h) tended to be lower than CW in CA. This suggested that the feeling of fatigue could be more rapidly reduced following CA trial and is therefore more beneficial in reducing the feeling of fatigue than CW during the short term recovery following 90 min running at 70% VO_2max_. In combination with other measurements, feeling of fatigue was positively correlated with the concentration of IL-6, cortisol, and muscle pain. It was negatively correlated with PPT (Table 4). With a similar response in acute muscle damage (as indicated by IL-6) and resting muscle pain, the attenuation of fatigue was associated with decreasing pain sensitivity following CA ingestion. Although there was no treatment effect in other mood dimensions and in the total score of mood disturbance, the attenuation of fatigue indicated a potentially enhancement of mood state. As a result, salivary cortisol, which suggests the HPA axis response to stress and is also considered as an indicator of negative affect (Buchanan et al., 1999), was also found to be reduced 4-h following exercise (Post-ex4h) with the attenuation of negative mood dimension of fatigue following the ingestion of CA. It can also be speculated that, compared with other mood dimensions, attenuation of fatigue should be considered as the primary goal during short term recovery following strenuous prolonged exercise. This goal can be achieved by the attenuation of muscle damage, reducing muscle pain, and pain sensitivity.

In summary, this study compared the effect of whey protein and alpha-lactalbumin on muscle damage, PPT, and mood states during short term recovery following strenuous prolonged exercise. Compared with the co-ingestion of CHO and whey PRO isolate, co-ingestion of CHO, and alpha-lactalbumin reduced sensitivity to the muscle pain was more beneficial in reducing the feeling of fatigue and cortisol responses during 4-h recovery following 90 min running at 70% VO_2max_.

## Author contributions

LQ and SW were responsible for designing those studies, conducting data analysis, interpreting the results, preparing for the submission, and revision of the manuscript. FS offered intellectual contributions in study design, data analysis, and manuscript preparation. YH offered his expertise in human physiology area and organized the plasma/salivary sample evaluation. SS offered contributions in manuscript preparation and English proofreading. CS provided her expertise in interpreting the experiment results of psychological measurements.

### Conflict of interest statement

The authors declare that the research was conducted in the absence of any commercial or financial relationships that could be construed as a potential conflict of interest. The handling editor and reviewer LA declared their involvement as co-editors in the Research Topic, and confirm the absence of any other collaboration.
